# Tiotropium in asthma: what is the evidence and how does it fit in?

**DOI:** 10.1186/s40413-016-0119-y

**Published:** 2016-09-14

**Authors:** David M. G. Halpin

**Affiliations:** Royal Devon & Exeter Hospital, Exeter, EX2 5DW UK

**Keywords:** Asthma, Long-acting anticholinerics, Tiotropium

## Abstract

Despite current therapeutic approaches asthma remains uncontrolled in a significant proportion of patients. Short-acting anticholinergic bronchodilators have a very long history of use in asthma, and recent data confirms the importance of acetylcholine as both a bronchoconstrictor and as a regulator of inflammation and remodeling in the lungs. Data from a comprehensive clinical trial programme, as well as use in primary care, show the efficacy and safety of tiotropium in adults with mild to moderate asthma when it is added to ICS and in severe asthma when it is added to high doses of ICS plus LABA, as well as in adolescents. Tiotropium is cost effective and its benefits are not restricted to particular phenotypes, making it a useful addition to the therapeutic options recommended by the Global Initiative for Asthma (GINA) for people with poorly controlled asthma at steps 4 & 5.

## Background

Asthma remains a major challenge for individuals and heath service. It affects approximately 235 million people worldwide [1] and is reported to be the most common chronic disease in children, affecting 10 % of children aged 12–18 years in the USA [2]. Current guidelines emphasize the importance of effective treatment for achieving and maintaining control. Asthma control consists of 2 domains: current impairment or day-to-day asthma control (absence of symptoms, minimal reliever use, and normal activity levels and lung function) and control of future risk (absence of exacerbations, prevention of decline in lung function, and absence of side effects from drugs) [3, 4].

Despite major advances in the management of asthma made in the 1970s with the introduction of inhaled corticosteroids (ICS) and again in the 1980s with the introduction of long-acting beta agonists (LABA) [5]; and with the development of national and international management guidelines [1, 2], many patients, including children and adolescents remain uncontrolled and remain at risk of exacerbations [6–8]. In 2011, the Innovative Medicine Initiative (IMI) proposed that these patients should be divided into those with “difficult-to-control asthma” and those with “severe refractory asthma.” [9]. Difficult to control asthma may be due to a number of reasons including: confounding illnesses, incorrect choice of inhaler or poor inhaler technique, concurrent smoking, uncontrolled rhinitis and non-adherence, either intentional or unintentional [10, 11]. Whereas people with severe refractory asthma have poor asthma control or frequent severe exacerbations per year despite the prescription of high-intensity treatment having had compliance checked, alternative diagnoses excluded, comorbidities treated and trigger factors removed. They can often only maintain adequate control when taking systemic corticosteroids and as a result are at risk of serious adverse effects.

The prevalence of severe refractory asthma has previously been estimated to be around 5 % to 10 % of the total asthmatic population [12–14], but a more reliable recent accurate estimate puts it at around 4 % [15]. This is still a substantial number of patients: approximately 8 million worldwide if the estimates of prevalence are correct.

Tiotropium is an effective long-acting muscarinic antagonist (LAMA) bronchodilator that has been used in them management of COPD for over 10 years. This review discusses recent evidence about its efficacy and safety in asthma and discusses its role in clinical practice.

### The role of bronchodilators in asthma

The paradigm changing Formoterol and Corticosteroids Establishing Therapy (FACET) study showed that adding a long-acting bronchodilator to low dose inhaled steroids was more effective at achieving control than increasing the dose of ICS [16]. This led to a fundamental change in the stepwise approach to asthma management: recognizing the importance of effective bronchodilator therapy in achieving control. Subsequent studies have shown that LABA/ICS can achieve well-controlled asthma in around 70 % of patients but not all [17].

Over time, LABA therapy loses some of its efficacy, as a result of tachyphylaxis [18]. There is a small reduction in maximal bronchodilator effect, but more importantly a loss of bronchoprotection (i.e., suppression of induced bronchospasm) against inhaled methacholine or histamine [19]. This tachyphylaxis also leads to some loss of responsiveness to short-acting beta agonist reliever therapy [20, 21]. As a result of these findings, the US Food and Drug Administration (FDA) recommended that LABA should not be given for long term therapy even in combination with ICS, and that where possible patients should step down to ICS alone [22]. There was, therefore, a clear need for alternative therapies.

Given the concerns about beta agonists and the fact that patients remain uncontrolled despite LABA/ICS therapy there has been renewed interest in the use of anticholinergics. Anticholinergic bronchodilators are the oldest documented therapy for asthma [23]. Short acting muscarinic antagonists (SAMA) such as ipratropium have been widely used for many years and are effective in the management of acute severe asthma [24, 25]. However, they appear less effective in stable disease where they seem to add little additional bronchodilatation to regular short-acting beta agonists (SABA) or LABA [26–28].

The lack of benefit from SAMA was thought to be due to the fact that cholinergic tone had a small role in determining airway caliber in stable asthma. However evidence on the efficacy of tiotropium has suggested that this is not the case and that cholinergic activity is responsible for increased bronchial smooth muscle tone.

### Mechanisms of action of LAMA

Acetylcholine is synthesized from choline and acetyl-CoA mainly by the enzyme choline acetyltransferase which is expressed in parasympathetic neurons in the airways but also in airway epithelial cells [29–31]. Non-neuronal acetylcholine released from epithelial cells acts a paracrine/autocrine hormone maintaining cellular homeostasis and epithelial repair, regulating ciliary activity and mucociliary clearance and modulating the activity of inflammatory cells [32]. Acetylcholine exerts a pro-inflammatory effect by attracting inflammatory cells and promoting their survival and cytokine release [30].

Human airways contain M1, M2, and M3 muscarinic receptors [33]. M3 receptors mediate acetylcholine’s effects on airway smooth muscle tone and mucous secretion from mucosal glands. M2 receptors have an inhibitory auto-regulatory effect on the release of acetylcholine from both pre- and post-ganglionic nerve terminals but are also widely expressed by other cells such as fibroblasts and smooth muscle cells [34]. In allergic asthma eosinophil-derived major basic protein acts as an allosteric antagonist of the M2 receptor, leading to inhibition of the auto-inhibition of acetylcholine release. M1 receptors are expressed on post-ganglionic nerves in the ganglia and by airway epithelial cells, where they have a modulatory role in electrolyte and water secretion.

Both neuronal and non-neuronal acetylcholine regulate inflammation and remodeling via direct effects on M3 receptors airway cells, but also indirectly as a result of mechanical stress induced in the epithelium as a result of compression due to bronchoconstriction.

Tiotropium has a higher selectivity for M3 receptors than for M2 receptors, and dissociates more slowly from M3 receptors than from M2 receptors [35]. It’s effect on airflow obstruction is mediated by its antagonism of M3 receptors leading to airway smooth muscle relaxation. It also appears to have anti-inflammatory properties which are also due to M3 antagonism. In animal models pretreatment with tiotropium reduces eosinophilic inflammation in response to allergen exposure and partly prevents aspects of airway remodeling, inhibiting mucus gland hypertrophy and decreasing the number of MUC5AC-positive goblet cells, as well as reducing airway smooth muscle thickening [36, 37]. In animal models, tiotropium inhibits neutrophil chemotactic activity, and decreases levels of cytokines (IL-4, IL-5, IL-13 & TNF-α) and leukotriene B4 in bronchoalveolar lavage fluid [38, 39].

### Trials of tiotropium in asthma

#### Early studies

The effects of tiotropium in asthma were first explored over 20 years ago when the sustained bronchodilator effects and protective effects against methacholine challenge were shown [40]. Single doses of tiotropium caused sustained increases of approximately 10 % in forced expiratory volume in 1 s (FEV1) in people with baseline FEV1 values of at least 80 % predicted. The bronchoprotector effect was sustained for at least 48 h.

A small study subsequently in patients with severe asthma treated with salmeterol and ICS showed that adding tiotropium and halving the dose of ICS led to an improvement in lung function with no change in mini-Asthma Quality of Life Questionnaire (AQLQ) scores [41].

The National Heart, Lung, and Blood Institute Asthma Clinical Research Network (ACRN) in the USA undertook a study similar to the FACET study to investigate the effects of adding tiotropium to an inhaled glucocorticoid, compared to doubling of the dose of the inhaled glucocorticoid or adding the LABA salmeterol [42]. The patients were non-smokers (<10 pack years) with a history of asthma confirmed by bronchodilator reversibility or bronchial hyper-responsiveness, and an FEV1 > 40 % predicted. During the 4 week run-in phase all patients were treated with beclomethasone 80 μg twice daily. If patients had an FEV1 < 70 % predicted at the end of the run-in they were randomised to have tiotropium 18 μg from the Handihaler device every morning or salmeterol 50μg twice daily added or to receive beclomethasone 160 μg twice daily for 14 weeks. The study was double blind and placebo controlled.

Two hundred ten patients were enrolled. Tiotropium improved lung function and symptom control compared with doubling the ICS dose (mean difference between tiotropium and doubling the ICS dose in morning peak expiratory flow (PEF): 25.8 L/min, *p* < 0.001; evening PEF: 35.3 L/min, *p* < 0.001; pre-bronchodilator FEV1: 0.10 L, *p* = 0.004; seven-question ACQ (ACQ-7): −0.18, *p* = 0.02). Tiotropium was non-inferior to salmeterol for all outcomes but increased the pre-bronchodilator FEV1 more than did salmeterol, with a difference of 0.11 L (*P* = 0.003). A subsequent analysis showed that although approximately equal numbers of patients showed a positive response in PEF and asthma control days to tiotropium and salmeterol, more showed a positive response in FEV1 with tiotropium [43]. This analysis also showed that a positive lung function response to tiotropium (in both FEV1 and morning PEF) was apparent in patients with higher cholinergic tone, indicated by a lower resting heart rate, and increased airway obstruction.

A proof of concept study investigated the efficacy and safety of added tiotropium in inadequately controlled asthmatic patients (Asthma Control Questionnaire (ACQ) score >1.5 and post-bronchodilator FEV1 < 80 % of predicted) despite treatment with at least high-dose ICS plus LABA (GINA step 4–5). 107 patients were randomized in this double-blind, placebo-controlled, crossover study comprising three 8–week treatment periods. All patients received 5 or 10 μg of tiotropium or matching placebo administered once daily in the morning through the Respimat soft mist inhaler, in a random sequence. Both doses of tiotropium significantly increased peak FEV1 compared to placebo (5 μg difference, 0.139 L; 95 % CI, 0.096–0.181 L; 10 μg difference, 0.170 L; 95 % CI, 0.128–0.213 L) with no significant difference between the active doses. Other lung function secondary end points responses were consistent with the primary end point results: both doses of tiotropium increased the trough FEV1 at the end of the dosing interval (5 μg: 0.086 L [95 % CI, 0.041–0.132 L]; 10 μg: 0.113 mL [95 % CI, 0.067–0.159 mL]; both *P* < .0004) and daily home peak expiratory flow measurements were higher with both tiotropium doses. Both doses had sustained effects on FEV1 compared to placebo over a 24 h period in a subgroup of patients in whom this was assessed. There were only small and non-significant differences in rescue medication use among the 3 periods, and minimal not significant changes in mini–AQLQ scores over the entire treatment period of 0.1 points for both active treatments compared with placebo and no significant differences in the symptom scores measured with an electronic diary. Subgroup analyses based on sex, FEV1% predicted or reversibility at screening, smoking status, or asthma duration showed that the effects of both doses of tiotropium compared with placebo were not significantly affected by these factors. The incidence of adverse events was comparable between treatment groups, although dry mouth occurred at a higher incidence in patients treated with tiotropium 10 μg (7 % v 2 % with tiotropium 5 μg and 1 % with placebo).

A second proof of concept study examined the effect of tiotropium compared to salmeterol in 388 patients with B16-Arg/Arg beta-2 receptor polymorphism and moderate symptomatic asthma despite maintenance therapy with 400–1000 μg of budesonide or equivalent per day [44]. At the time these patients were thought to be less responsive to LABAs but the B16-Arg/Arg phenotype has subsequently been shown to have no influence on responses to salmeterol therapy in asthmatic patients.

During the run-in period all patients were treated with salmeterol 50 μg twice-daily via a metered-dose inhaler. They were then randomised to 16 weeks of treatment with tiotropium 5 mg via Respimat once daily in the evening, to continue salmeterol 50 μg twice daily, or placebo, whilst continuing their ICS. Mean weekly morning pre-dose PEF was maintained during the treatment period with tiotropium and salmeterol but decreased in patients switched to placebo. Tiotropium was non-inferior to salmeterol. The overall incidences and patterns of adverse events were similar across all groups.

The optimal treatment tiotropium regime in asthma was examined in two phase II studies. The first was a 4 week, 4 way cross over dose ranging study of tiotropium 5, 2.5 and 1.25 μg and placebo via the Respimat once-daily in the evening as an add-on to medium-dose ICS in 149 adults with moderate asthma [45]. Statistically significant improvements in peak FEV1 (0–3h) response and ACQ-7 scores were observed with each tiotropium dose compared with placebo. The largest improvements were with the 5 μg dose. Adverse events were comparable between treatment groups.

The second study compared once daily dosing with 5μg tiotropium in the evening with twice daily dosing with 2.5μg tiotropium in 94 adults with moderate asthma treated with moderate dose ICS (400–800 μg of budesonide or equivalent per day) over 4 weeks in a crossover study [46]. Both once-daily 5 μg and twice-daily 2.5 μg regimes provided significant and comparable 24-h bronchodilation (once-daily 5 μg, 0.158 L compared with placebo; twice-daily 2.5 μg, 0.149 L (both *p* < 0.01)), and similar improvements in peak FEV1, trough FEV1, and morning and evening PEF. Adverse events were again comparable between all treatment regimens.

#### Phase III studies in adults

The longer term efficacy of once daily tiotropium as add-on to at least ICS maintenance therapy in adult patients across all severities of symptomatic asthma has been investigated in five phase III double-blind, placebo-controlled, parallel-group trials. The two identical PrimoTinA trials investigated the effects of tiotropium 5 μg or placebo as an add-on to high-dose ICS (≥800 μg budesonide or the equivalent) plus a LABA over 48 weeks in 912 patients with severe symptomatic asthma [47]. All patients had an ACQ-7 score ≥ 1.5, a post-bronchodilator FEV1 ≤ 80 % predicted value and FEV1/FVC ≤ 70 % and had had at least one exacerbation that was treated with systemic glucocorticoids in the previous year. They were also either lifelong non-smokers or to have a smoking history of fewer than 10 pack-years, with no smoking in the year before the study. Tiotropium significantly improved peak FEV1 by a mean 0.110 L (*p* < 0.0001) and trough FEV1 by a mean of 0.093 L (*p* < 0.0001) and increased the time to both the first severe asthma exacerbation (hazard ratio 0.79, *p* = 0.03) and the first episode of asthma worsening (hazard ratio 0.69, *p* < 0.001). Improvements in asthma control and quality of life were also observed in both trials with marked improvements in the placebo groups particularly in trial 1. At week 24, the mean difference in ACQ-7 scores and AQLQ score between groups was only statistically significant in trial 2 but did not exceed the minimal clinically important differences for the ACQ-7 of 0.5 [48] or AQLQ of 0.5 [49]. The incidence of adverse events was comparable between treatment groups.

Pre-specified post hoc subgroup analysis showed that the improvements in peak FEV1 in the tiotropium tended to be higher in patients with a lower FEV1 and in ex-smokers, but were not related to level of reversibility, age, body-mass index, allergic status, asthma duration, ACQ-7 score at baseline, or prior use of systemic glucocorticoids.

A further two replicate, double-dummy trials have examined the effects of tiotropium in 2103 patients with moderate symptomatic asthma (ACQ-7 score ≥ 1.5) and persistent airflow obstruction (post-bronchodilator FEV1 60–90 %) despite treatment with medium-dose ICS (≥800 μg of budesonide or the equivalent) [50]. Patients received once-daily tiotropium 5 or 2.5 μg, twice-daily salmeterol 50 μg, or matching placebo for 24 weeks. Both doses of tiotropium significantly improved lung function compared with placebo (pooled effect in peak FEV1 0.185 L (95 % CI 0.146–0.223 L; *p* < 0.0001) with 5 μg; 0.223 L (95 % CI 0.185–0.262 L; *p* < 0.0001) with 2.5 μg), with the improvements in peak FEV1 maintained over 24 h. Both doses also led to significant improvements in asthma control, as assessed by ACQ-7 responder rate (5 μg: OR 1.32, 95 % CI 1.02–1.71, *p* = 0.035; 2.5 μg: OR 1.33, 95 % CI 1.03–1.72, *p* = 0.031), and the risk of first severe asthma exacerbation or first episode of asthma worsening was reduced with tiotropium add-on therapy. The effects of tiotropium were similar to those of salmeterol which increased peak FEV1 by 0.196 L (95 % CI 0.158–0.234 L; *p* < 0.0001) and increased the proportion showing an ACQ response (OR 1.46, 95 % CI 1.13–1.89, *p* = 0.0039). The incidence of adverse events was similar across all treatment groups.

To complete the phase III programme in adults a further trial examined the effect of tiotropium in adults with symptomatic asthma (ACQ-7 score ≥ 1.5) and persistent airflow obstruction (post-bronchodilator FEV1 60-90 %) on low- to medium dose ICS (200–400 μg of budesonide or the equivalent) therapy [51]. 464 patients were randomised to 12 weeks treatment with 2.5 μg or 5 μg tiotropium or placebo once daily in the evening via Respimat. Both doses of tiotropium improved peak FEV1 (0–3h) compared to placebo (adjusted mean difference 0.128 L 95 % CI 0.057–0.199 L, *p* < 0.001 for 5 μg; 0.159 L 95 % CI 0.088–0.230 L, *p* < 0.001 for 2.5 μg). Both doses also improved the secondary endpoints of trough FEV1 and FEV1 area under the curve (0–3h), and morning and evening PEF compared to placebo. The incidence of adverse events was similar across all treatment groups.

A one year safety study of tiotropium in asthma has been carried out in Japan [52]. 285 patients were randomised to receive 2.5 μg or 5 μg tiotropium or placebo once daily in addition to LABA/ICS. There was no significant difference in adverse events between the groups.

#### Phase III studies in adolescents and children

Following on from the studies in adults, studies of the effects of tiotropium in adolescents and children with asthma have been undertaken. Phase II studies in adolescents showed similar effects on lung function when tiotropium is added to LABA/ICS as those seen in adults [53, 54]. A 48 week phase III study investigated the effects of adding 2.5 μg or 5 μg tiotropium or placebo once daily in 398 adolescents (age 12–17) with symptomatic asthma (ACQ-7 score ≥ 1.5) and a pre-bronchodilator FEV1 60–90 %, despite therapy with ICS with or without a LABA or leukotriene receptor antagonist (LTRA) [55]. Peak FEV1(0–3h) was statistically significantly greater with both doses of tiotropium (0.174 L 95 % CI 0.076–0.272 L, *p* < 0.001 for 5 μg; 0.134 L 95 % CI 0.034–0.234 L, *p* < 0.01 for 2.5 μg) and trough FEV1 was also statistically significantly improved by 5 μg (0.117 L 95 % CI 0.010–0.223 L, *p* < 0.03) but not by 2.5 μg. Weekly mean morning and evening PEF were also improved and there was a trend for an improvement in asthma control and quality of life.

A 12 week study in China compared ICS to ICS plus tiotropium in 80 children aged 6–14 with newly diagnosed moderate persistent asthma [56]. Compared with baseline, FEV1, FVC and PEF were significantly improved in both groups at 4, 8, and 12 weeks (*P* < 0.01) with significantly larger improvements in the tiotropium group compared with the control group (*P* < 0.05). Rescue medication use and night time waking were significantly reduced in the tiotropium group compared to the control group (*P* < 0.05; *P* < 0.001 respectively). Further studies in adolescents, children aged 6-11 and infants aged 1-5 have been completed.

#### Real world studies and health economic analyses

Although unlicensed, long-acting anti-muscarinics have been prescribed for the treatment of asthma in the United Kingdom (UK) since 2002, predominantly as an add-on therapy in older patients with poorly controlled asthma despite good treatment compliance and usually on the recommendation of specialists. A retrospective study of data from a UK primary care database on 2042 adults prescribed tiotropium with a diagnosis of asthma but no diagnosis of chronic obstructive pulmonary disease (COPD) patients showed that in the 12 months after the addition of tiotropium compared to the pervious 12 months there was a significant decrease in the number of people having exacerbations (26.6 % v 36.8 %, *p* < 0.001) and course of oral steroids (25.5 % v 35.8 %, *P* < 0.001), and a significant increase in the number of people with controlled asthma (52.4 % v 41.4 % *p* < 0.001) [57].

An economic model has been developed to look at the cost-effectiveness of tiotropium add-on therapy, from the perspective of the UK National Health Service [58]. Using data from the paired PrimoTinA trials [47] this model found that in patients with severe symptomatic asthma despite treatment with high-dose ICS plus LABA, add-on tiotropium therapy was cost effective. It generated an incremental 0.24 quality adjusted life years (QALY) and £5,238 costs over a lifetime horizon, resulting in an incremental cost-effectiveness ratio of £21,906 per QALY gained.

### What does this mean for clinical practice ?

These trials in adults show the efficacy and safety of tiotropium in people with mild to moderate asthma when it was added to ICS and in people with severe asthma when it was added to high doses of ICS plus LABA, as well as in adolescents. Early studies of other LAMAs (glycopyrrolate and umeclidinium) in asthma have been published [59, 60], but tiotropium is currently the only LAMA licenced for use in asthma.

Adding tiotropium to ICS in people with uncontrolled mild to moderate asthma is safe and effective and the efficacy of tiotropium is similar to that of salmeterol. In time adding a LAMA may become the recommended option given the concerns about tachyphylaxis to LABAs but the lack of a combination inhaler containing both a LAMA and an ICS may limit the uptake of this approach as there would be real concerns that patients may omit the ICS and rely on the symptomatic benefit of the LAMA leaving them at significant risk of exacerbations and even death as was seen when LABA inhalers were first introduced [61, 62].

The biggest unmet need is in people with uncontrolled asthma despite treatment with ICS (whether medium or high dose) plus LABA. In this group tiotropium significantly improved lung function and reduced the frequency of exacerbations [47]. The effect is not dependent on the asthma phenotype and appears cost effective, making this a clinically very useful option for these patients. Real world data confirms the efficacy of tiotropium in asthma beyond the constraints of a randomised clinical trial. On the basis of this evidence the GINA report now recommends tiotropium as an add on therapy at steps 4 and 5 [4], and as more evidence emerges and experience grows it may become the preferred option.

## Conclusions

Tiotropium appears to be safe, effective and cost effective in people with uncontrolled severe asthma when added to high doses of ICS plus LABA. This group of patients has the biggest unmet need for additional therapy and tiotropium appears equally effective in all phenotypes. Tiotropium is also safe and effective in adults with mild to moderate asthma when added to ICS, and in adolescents. Data from on-going studies will clarify its role in children. It is currently the only LAMA licenced for use in asthma and is now recommended as an add on therapy at steps 4 and 5 in the GINA report.

## Abbreviations

ACQ, asthma control questionnaire; ACRN, asthma clinical research network; AQLQ, asthma quality of life questionnaire; COPD, chronic obstructive pulmonary disease; FACET, formoterol and corticosteroids establishing therapy; FDA, US food and drug administration; FEV1, forced expiratory volume in 1 s; GINA, Global initiative for asthma; GOLD, Global obstructive lung disease initiative; ICS, inhaled corticosteroids; IL-13, Interleukin 13; IL-4, Interleukin 4; IL-5, Interleukin 5; IMI, innovative medicine initiative; LABA, long-acting beta agonists; LAMA, long-acting muscarinic antagonist; LTRA, leukotriene receptor antagonist; PEF, peak expiratory flow; QALY, quality adjusted life years; SABA, short-acting beta agonist; SAMA, short-acting muscarinic antagonist; TNF-α, tumor necrosis factor alpha; UK, United Kingdom
